# Digital Occlusion Tracking by T-Scan Novus System in Bruxism Patients Treated With 3D Printed Occlusal Splints

**DOI:** 10.1155/ijod/8842498

**Published:** 2025-04-22

**Authors:** Dobromira Shopova

**Affiliations:** Department of Prosthetic Dentistry, Faculty of Dental Medicine, Medical University, Plovdiv, Bulgaria

**Keywords:** 3DISK OVO, 3Shape, bruxism, dentistry, digital occlusion, intraoral scanning, T-scan novus

## Abstract

**Background:** Bruxism is a chronic pathological condition with significant clinical implications, necessitating meticulous monitoring for comprehensive treatment. The primary aim of this study was to conduct a digital measurement-based comparative analysis of occlusal alterations in occlusal splints over a 3-month period.

**Methods:** This investigation involved 32 patients with natural dentition, fixed dental restorations, implant treatments, and a minimum of second molars. Intraoral scanning was executed using the 3DISK OVO imaging system. Digital models generated from these scans were employed to design occlusal splints using the 3Shape design software in conjunction with its splint studio module. The splints were subsequently fabricated using 3D printing technology and a biocompatible resin, Ortho Rigid (Nextdent). The assessment of occlusion was carried out using the T-Scan Novus system (Tekscan, 2018) and subjected to analysis with licensed software version 10.0.40 (T-Scan 10). Student's *t*-test for independent samples and a paired-sample *t*-test were used to detect the statistically significant difference in the distribution of occlusal force.

**Results:** Initial digital occlusal measurements revealed statistically significant disparities in two specific regions between male and female subjects—the right first molar (*t*(31) = 2.04, *p* < 0.05) and left second molar (*t*(31) = 1.95, *p* < 0.05). Following a 3-month follow-up, significant differences in occlusal splint wear were discerned among male subjects (*p* < 0.01), whereas such differences were not observed among female subjects (*p* > 0.01).

**Conclusion:** The digital design of occlusal splints facilitates the development of uniform contact areas across the entire occlusal surface. Nonetheless, the examination with T-Scan Novus unveiled that uniformity in contact area does not necessarily correspond to uniformity in force distribution.

## 1. Introduction

Digital technology is not a dream in dentistry. Intraoral scanners (IOSs), pivotal tools enabling the acquisition of digital dental impressions, confer a diverse array of advantages and applications. These benefits include the elimination of direct contact with oral mucosa, thereby enhancing patient comfort by obviating the requirement for conventional impression materials. Moreover, they afford instantaneous visual display on screens, facilitating inspection and enabling immediate corrections. Real-time visualization capabilities, coupled with their inherent time efficiency, further underscore their utility. Additionally, IOSs offer the convenience of repeated scanning processes with ease and selectivity, augmenting their versatility and effectiveness in clinical practice [1]. Nonetheless, the precision of IOSs can be influenced by various factors, including variations in dental arch width arising from individual characteristics such as facial morphology, head size, and age (child or adult), which may impact the accuracy of full jaw scans [2, 3]. Accuracy in intraoral scanning comprises two essential and complementary elements: “trueness” and “precision.” Trueness pertains to the measurement's capability to accurately correspond to the actual value of the quantity being measured, while precision relates to the scanner's ability to produce consistent and reproducible results when measuring the same object under varying conditions [4]. The continuous evolution of IOSs has led to the incorporation of supplementary functionalities, including their application as detection systems for oral cavity pathologies. These IOS applications hold particular significance in the field of oral health diagnostics, covering areas such as the identification of dental caries, dental wear, periodontal diseases, and oral cancer [5].

A variety of occlusal analyzers are employed to evaluate occlusal–articulation relationships. These analyzers can be broadly categorized as qualitative or quantitative indicators, with the principal distinction lying in the capability of quantitative indicators to measure tooth contact events. Qualitative indicators consist of tools such as articulating paper, articulating silk, occlusal spray, articulating film, metallic shim stock film, and high spot indicators. In contrast, quantitative indicators encompass the T-Scan occlusal analysis system and the Virtual dental patient [6, 7]. Articulation paper is the most commonly used clinical method for examining and visualizing occlusal contacts. However, this conventional method carries a potential for errors, as extensive plane contacts do not necessarily indicate strong contacts. In practice, it has been demonstrated that single-point contacts are more reliable [8]. Accurately measuring bite force is pivotal in evaluating the health of the masticatory system, yet only a limited number of commercially available transducers have undergone validation for routine clinical use. The T-Scan Novus serves as an invaluable objective assessment tool for analyzing a patient's occlusion. Unlike traditional articulating paper, which merely identifies contact locations, the T-Scan Novus distinguishes both force and timing, two fundamental parameters essential for comprehensive occlusal measurement [9]. Through the T-Scan occlusal analysis system, clinicians can capture bite force distribution, providing insights into relative intensity and occlusal timing [10]. It offers various modes for recording and analyzing occlusal contacts, including the balance plot, time display, and comparison screen [11]. The most recent iteration of the T-Scan system, introduced in 2018, is T-Scan 10, which boasts enhanced software features that augment the clinician's capacity to assess occlusal function. These features encompass an overloaded Implant Warning tool, a Force Eraser designed to eliminate sensor artifacts when the sensor is folded between or around overlapping anterior teeth, and the Digital Impression Overlay, enabling superimposition of T-Scan data onto an STL digital arch scan [12].

Bruxism, a common parafunctional habit, has a complex etiology involving biological, physiological, and external factors. It can significantly impact quality of life, leading to dental issues like tooth wear, fractures of dental restorations, and orofacial pain. Diagnosis often relies on observable symptoms such as muscle fatigue, TMJ pain, ear discomfort, tooth soreness, or a sensation of loose teeth. However, there is currently no universally accepted clinical method for assessing bruxism that is both diagnostically reliable and cost-effective [13].

Bruxism is classified into two main types: static bruxism, involving compression without lateral movement, and dynamic bruxism, characterized by both compression and horizontal movements. It can occur during the day (as static clenching) and at night (as dynamic grinding) [14]. The consequences of bruxism are varied and may present as temporomandibular joint (TMJ) pain and dysfunction, head and neck pain, tooth wear, tooth mobility, erosion, abrasion, damage to supporting structures, muscle pain and spasms, aesthetic disturbances, and oral discomfort [15]. Noncarious destructive processes, such as attrition, abrasion, and erosion, are commonly observed in individuals with bruxism, resulting in adverse effects on the teeth [16]. Abrasion wear is predominantly attributed to bruxism and excessive toothpaste use, while erosion wear can be linked to factors like regurgitation, consumption of acidic beverages like cola, and fruit consumption [17].

A splint is a commonly employed treatment method for managing bruxism. As per the Glossary of Orthodontic Terms, a splint refers to a range of apparatus, appliances, or devices utilized to stabilize or support teeth or bones and to resist motion or displacement of fractured or injured structures [18]. The stabilization splint is often prescribed to alleviate symptoms associated with masticatory dysfunction, including muscle pain, TMJ pain, clicking, crepitation, restricted movement, and coordination issues. This type should be worn constantly, except during meals, and can be placed on either the upper or lower jaw [19].

The primary role of the splint is to function as a stress reliever, dispersing the additional stresses generated and diminishing deformities and deviations in the temporomandibular system (TMS) brought about by bruxism. The splint facilitates bilateral and simultaneous loading, aiding in the regulation of bruxism by establishing a biomechanical balance between the physiological load and the generated stress [20]. In patients with bruxism, abnormal contractions of the masseter muscles can result in myofascial pain. Research suggests that the use of an occlusal splint reduces the hyperemic response, indicating a reduction in the force of masticatory muscle contraction [21]. Additionally, another study found that splint therapy significantly reduced masseter muscle thickness and improved elasticity [22].

However, as of a literature review conducted in 2021, there remains insufficient evidence to conclusively establish whether occlusal splint therapy for bruxism offers superior benefits compared with no treatment, alternative oral appliances, behavioral interventions, or pharmacological therapy. Moreover, the existing studies are burdened with limitations and possess a high risk of bias. Further research is imperative in this domain to enhance the quality of clinical trials and attain more definitive conclusions [23].

In contemporary dentistry, digital workflows have become well-established, facilitating the implementation of entirely digital protocols. Crucial steps in this process encompass digital impressions and the determination of the centric relation position. After proper digital design, the splint can be manufactured using 3D printing or CAD/CAM technology [24, 25]. For individuals with awake bruxism, smartphone applications have proven to be valuable tools for identifying 70% of symptoms through the analysis of various behavioral frequencies. These technological solutions have gained popularity among both younger and older populations due to their efficiency, user-friendliness, and the capacity to effectively reduce deviations in assessment time [26].

The central objective of this article was to conduct a comparative analysis of occlusal changes in occlusal splints using digital measurements over a 3-month period.

## 2. Materials and Methods

The study involved a total of 32 participants, comprising 14 males and 18 females. Inclusion criteria for participation consisted of patients with natural dentition, fixed restorations, previous implant treatment, and the presence of at least their second molars. Exclusion criteria encompassed edentulous patients, those with removable prosthesis, and individuals with mobile teeth. The age range of the participants spanned from 23 to 55 years, with a mean age of 39.89 ± 5.14 years. Specifically, the average age for male participants was 41.56 (±3.61) years, while female participants had an average age of 36.45 (±6.27) years. A detailed breakdown of the patient demographics can be found in Table 1.

## 3. Study Design

For the intraoral scanning of both the upper and lower jaws, the 3 DISK OVO imaging system (3DISK, 2023) was employed [27]. The digital models derived from these scans were subsequently utilized in the 3Shape design software, specifically employing its splint studio module, to create the occlusal splint. These splints were then fabricated for the upper jaw utilizing 3D printing technology. The selected material for the splints was Ortho Rigid (Nextdent), a biocompatible resin that ensures a safe and comfortable fit for the patient. It is worth noting that this resin has a light blue color, which may be preferable for patients wearing it during nighttime use [28].

**Figure 1:**
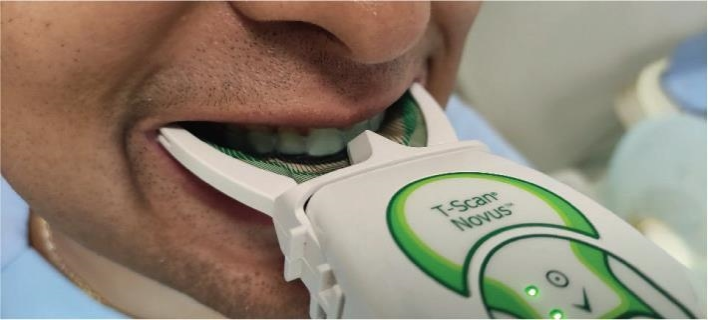
T-Scan Novus handpiece positioning with occlusal splint on the upper jaw.

The assessment of occlusion in this study utilized the T-Scan Novus system (Tekscan, 2018). This system involves the use of a pressure-sensitive plate positioned between the dental arches, with a marker pin placed between the upper central incisors. In this specific case, due to the presence of the splint, the marker was adjusted forward by 1.5 mm (vestibular thickness of the splint), as illustrated in Figure 1. The patient was positioned in an upright posture. This upright position allows for the relaxation of the mandibular opener muscles situated in the neck area and prevents any distalization (backward movement) of the mandible from its normal function [29]. It is important to note that in bruxists, mandibular levator muscles tend to exhibit higher activity compared with openers [30, 31]. The marker handle was placed horizontally, and the patient was instructed to close their teeth firmly. Due to the nature of the digital design (raising the antagonist cusp tips), the closure occurred in a single position.

This is the position where the upper and lower teeth come together in a stable and comfortable manner when the jaw is in a relaxed and natural resting state. It serves as the reference point for evaluating and adjusting occlusion or the way the teeth come together when the jaws close [32]. Three consecutive measurements were taken, with the first measurement serving as a training trial to ensure the patient's correct performance. The subsequent two measurements were employed for analysis, and the arithmetic average of these two consecutive measurements was calculated for each patient.

To ensure a precise comparison of the patients' digital occlusion, the following methodology was employed.

• **Image integration:** The intraoral images acquired through the 3 DISK OVO scanner were seamlessly imported into the T-Scan system, ensuring compatibility for subsequent analysis.
• **Specialized software:** The data analysis was conducted using the licensed software version 10.0.40, known as T-Scan 10. This software is specifically tailored for detailed occlusal analysis, providing advanced tools and features.
• **Quadrant division and force distribution calculation:** The dental arches were methodically divided into quadrants using the T-Scan 10 software. This division allowed for a structured and organized approach to the assessment. The software automatically computed the percentage distribution of occlusal forces within each individual tooth. This analysis primarily focused on comprehending how the forces were distributed during maximum intercuspidation, with the exclusion of wisdom teeth from this assessment.

By employing this comprehensive approach, the study was to offer precise insights into the distribution of occlusal forces during maximum intercuspidation for each tooth in the dental arches of the patients. This method facilitated a thorough comparison of digital occlusion patterns among the study participants.

### 3.1. Statistical Analysis

Descriptive statistics were used to assess the demographic and clinical characteristics of the study group. Categorical variables were presented as counts and percentages. Quantitative data were presented as mean ± SD. A Student's *t*-test for independent samples and a paired-sample *t*-test were used to examine whether there was a statistically significant difference in the distribution of occlusal force at the beginning and 3 months after occlusal splint placement, both between men and women and within each group respectively. The statistical analysis was performed using SPSS statistical software, version 23.

## 4. Results

**Figure 2:**
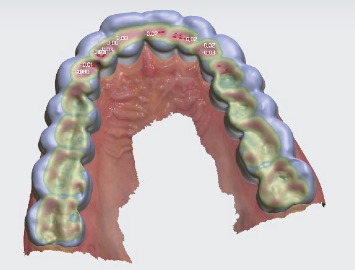
Digital design “raise to antagonist cusp tips”.

**Figure 3:**
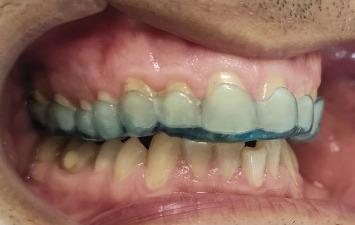
Intraoral view of the occlusal splint.

The digital design of the occlusal splint was executed through the utilization of the splint studio module within the 3Shape software. In this module, the initial option available for creating a stabilizing type of splint involved the elevation of antagonist cusp tips. The occlusal design was meticulously customized to align with the contours of the antagonistic teeth, guaranteeing a consistent position of the lower teeth during the closing phase. For a visual representation of this design process, Figures 2 and 3 illustrate the digital design and an intraoral view, respectively.

The vestibular and palatal thickness of the occlusal splints was standardized at 1.5 mm, as per the recommendations of the manufacturer. However, the occlusal thickness of the splints exhibited variation, ranging between 1.5 and 3.5 mm. This variation was influenced by individual characteristics such as abrasion, attrition, erosion, underbite, and other factors. It is worth noting that there was no statistically significant difference in splint thickness based on gender.

To monitor the usage of the splints, patients were requested to record the number of hours they wore the splint daily by means of a questionnaire. The average duration of splint usage was determined to be 6.28 ± 1.08 h for male participants and 7.14 ± 1.36 h for female participants. Notably, two female patients reported wearing the splint for an average of over 12 h per day due to their perception of daily passive clenching and wearing the splint during the day as well. Consequently, a statistically significant difference in wearing time between males and females was observed. Further information regarding the duration of splint usage can be found in Table 2.

Following the placement of the occlusal splint on the upper dental arch, a digital measurement of occlusion was conducted using the T-Scan Novus system. The T-Scan system generates a color map based on the pressure data collected by the sensor, indicating the strength of contacts between the splint and the lower teeth. The color-coded markings on the map signify contact strength, with strong contacts displayed in violet and red, followed by yellow, green, and weak contacts depicted in blue. By integrating the intraoral images obtained with the Trios color scanner into the software, the contacts can be precisely positioned on the tooth surfaces, ensuring their accurate alignment. This fusion of data from the intraoral images and the T-Scan system enables a more precise analysis of occlusion and force distribution in the dental arches. The force distribution on each tooth is presented as a percentage.

**Figure 4:**
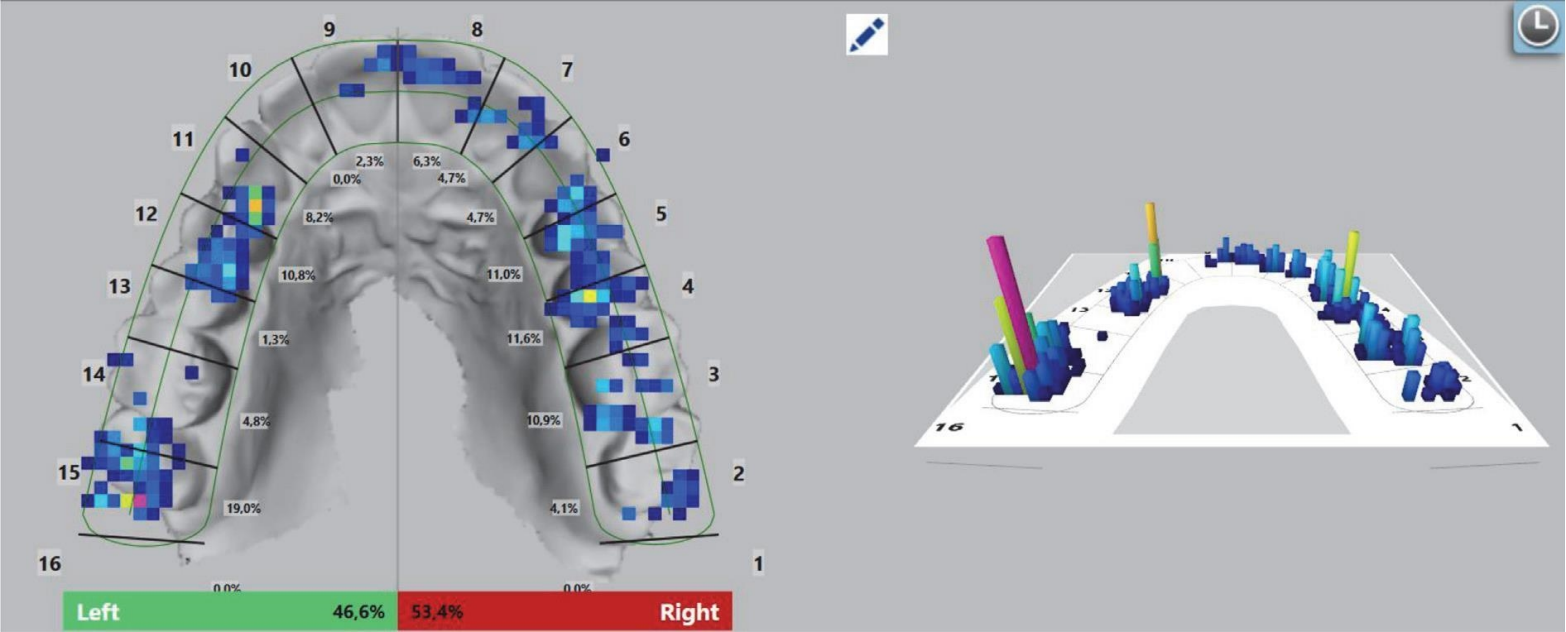
Force distribution on an average patient at the initial moment in the occlusal splint placement.

**Figure 5:**
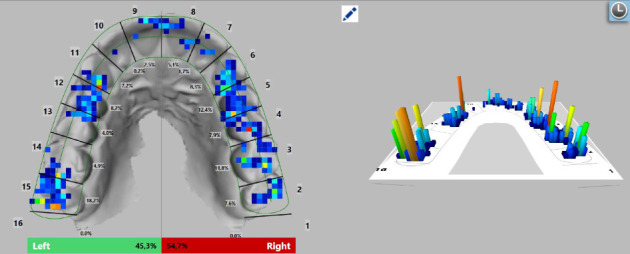
Force distribution on the same average patient 3 months after the occlusal splint placement.

To enhance the accuracy of the study, the force distribution of each tooth was measured and subsequently compared with a follow-up measurement conducted 3 months after the initial assessment. The T-Scan system employs the American dental formula for permanent dentition, with tooth numbers ranging from 1 to 16 for the upper jaw (right to left) and from 17 to 32 for the lower jaw (left to right) [33]. The variations in force redistribution are illustrated in Figures 4 and 5.

The digital measurement of occlusion provided data on the pressure distribution across each individual tooth, quantified as a percentage. Initial results revealed a statistically significant difference in force distribution specifically at the right first molar and left second molar between men and women (*p* < 0.05). However, the follow-up measurements conducted after 3 months showed no statistically significant differences between the genders in these areas (*p* > 0.01).

The results of both measurements are presented in Tables 3 and 4.

When the pressure data for each tooth in both the left and right halves were aggregated and compared between the baseline and the 3-month follow-up, a statistically significant difference between men and women was observed only at the 3-month interval (*p* < 0.05), as shown in Table 5.

When the aggregated data for both the left and right halves of the dentition were compared with men and women, a significant difference was detected in men (*p* < 0.01), but not in women (*p* > 0.01), as illustarted in Tables 6 and 7.

## 5. Discussion

Bruxism significantly impacts prosthetic outcomes, with occlusal splints being a primary intervention. Mandibular advancement devices and maxillary splints enhance sleep quality and mitigate sleep bruxism. Studies indicate that hard, soft, and nonoccluding splints improve symptoms within 90 days while reducing masticatory muscle tension [34–38].

With advancements in technology, both clinical and laboratory protocols can now seamlessly transition to a digital format. This includes steps like intraoral scanning, precise vertical registration, digital design, and fabrication using 3D printing. Additionally, occlusal splints can be made with CAD/CAM technologies, providing enhanced precision and customization, revolutionizing modern dental practice [25, 39, 40]. The use of modified centric relation recording allows for more accurate determination of occlusion by virtually aligning the maxillary and mandibular arches in both centric relation and the desired vertical dimension of occlusion [41]. Another modern method involves determining the centric relation (CR) position using the Luci Jig deprogrammer and utilizing a digital facebow to measure individual values [24].

Dimova-Gabrovska's study employed a four-stage algorithm with the T-Scan 8 system to analyze static, dynamic, and functional occlusion in patients with bruxism and bruxomania. The results revealed an absence of stable occlusion and a high prevalence of occlusal interferences, affecting dynamic and functional occlusion parameters in the study group [42].

In the investigation, the material utilized for 3D printing the occlusal splints was Ortho Rigid–NextDent. According to the manufacturer, this material exhibits favorable mechanical strength characteristics, including an ultimate flexural strength of ≥50 MPa, a flexural modulus of ≥1500 MPa, a maximum stress intensity factor of ≥1.1 MPa m1/2, and a total fracture work of ≥250 J/m^2^ [28]. In a comparative study evaluating conventional and 3D printed splints, no statistically significant differences were observed in linear measurements [43]. However, it is important to note that clinical studies on the durability and oral behavior of 3D printed splints are limited. Consequently, our understanding primarily relies on information provided by the manufacturer. In a study by Santos et al.[44], acrylic resin splints (both chemically and thermally activated) were compared with 3D printed splints. The resin used in their research exhibited inferior performance, particularly under thermal cycling conditions. Therefore, the study suggests that these splints should be used temporarily and for limited durations [44].

Bruxism is predominantly a unilateral movement that causes uneven effects on the temporomandibular system, muscles, and tooth surfaces. In contrast, bilateral clenching generates bite forces that are over 30% greater than those produced during unilateral clenching. While muscle activity in the masseter muscles remains symmetrical during unilateral clenching, the temporal muscles exhibit asymmetrical activation [45].

The masseter muscle plays a crucial role in the masticatory process. Its activation dynamically adjusts during natural chewing, especially during jaw closure. This adjustment is influenced by both early and late muscle activation components. Additionally, the modulation of jaw movements in response to food hardness is closely tied to the level of muscle activity observed during chewing [46].

In the context of temporomandibular disorders (TMD), the duration of masseter muscle electromyography (EMG) activity during sleep was found to be significantly associated with gender and joint sound scores. Other independent variables did not exhibit significant relationships with muscle activity variables. This suggests that both gender and joint clicking are related to the duration of masseter EMG activity during sleep [47]. In patients with myogenous craniomandibular disorder, researchers examined the immediate impact of a stabilization splint on the symmetry of masseter and anterior temporal muscle activity during submaximal clenching at five different levels using electromyography. Results showed that unbalanced splints led to a slight but statistically significant decrease in masseter muscle symmetry at the 10% clenching level (*p* < 0.01), while no such effect was observed in the temporal muscle. These findings suggest that monitoring masseter muscle symmetry can serve as an objective measure for assessing treatment effectiveness [48]. Surface electromyographic (EMG) recordings were obtained from the bilateral masseter and anterior temporal muscles during both daytime and sleep. Patients exhibited significantly higher asymmetry indices for the anterior temporal muscle during both daytime and sleep, while the masseter muscle showed significantly lower asymmetry during sleep compared with controls. These findings suggest a strong association with occlusal interference, instability due to malocclusion, and lateral mandibular deviation [49].

Ultrasonography presents diagnostic capabilities for assessing the masseter muscle in both normal and pathological conditions [50]. Additionally, thermographic evaluation offers another method for identifying alterations in the muscles and TMJ associated with temporomandibular disorders resulting from bruxism [51].

Different fiber type properties in the masseter muscle are linked to dentofacial deformities, particularly those involving vertical facial growth. Increased type II fiber areas inversely correlate with vertical facial dimensions, highlighting the association between muscle composition and facial morphology [52]. Women often report headaches and masseter muscle pain, especially with bruxism. In contrast, men exhibit increased muscle tension and stiffness during contraction. These differences underscore gender-specific variations in masseter muscle characteristics and their clinical implications [53]. Studies on children reveal a positive correlation between masseter thickness and mechanical advantage, as well as with the posterior-to-anterior facial height ratio. Gender differences exist, with females showing correlations between masseter thickness and facial angles [54]. Females tend to have greater maxillary intermolar width and masseter volume, particularly in low- and normal-angle subjects. Correlations exist between maxillary width and masseter volume, emphasizing gender and facial pattern influences on orthodontic assessments [55].

Normative values of the masseter reflex are limited, but a study involving 105 healthy volunteers aged 5–78 years shed light on this aspect. The study found a positive correlation between age and latencies and a negative correlation between age and amplitude of the masseter reflex. The masseter reflex appears to be a reliable and robust clinical test when age and gender differences are taken into account [56].

The Salah's study aimed to investigate the correlation between bruxism and facial coordination in individuals with awake or sleep bruxism. Participants underwent video recording to assess facial symmetry during various movements, including raising eyebrows and tight eye closure for upper face evaluation, as well as blowing and smiling for lower face assessment. Results revealed a statistically significant difference in movement distances between the right and left sides of the face. Additionally, significant differences were observed in the mean values of all movement distances, except for eyebrow raising, and in the mean duration of all movements. Findings suggest that bruxism primarily affects bilateral coordination of lower facial movements rather than upper facial movements [57].

## 6. Conclusions

The digital design of occlusal splints enables precise customization, ensuring uniform contact across the occlusal surface to promote a balanced bite and minimize uneven stress, crucial for bruxism management. A study revealed gender-related differences in occlusal adaptation, with statistically significant variations in the right first molar and left second molar between men and women. These findings suggest gender may influence splint effectiveness. Further research is needed to explore the long-term impact of occlusal splints, enhancing individualized treatment strategies for bruxism and related orofacial conditions.

## Figures and Tables

**Table 1:** Demographic and clinical characteristics of the patients included in the study.

Demographic and clinical characteristics	Subjects, *N* = 32 *n* (%)
Age (years)	39.89 ± 5.14*⁣*^*∗*^
Male	14 (43.75%)
Female	18 (56.25%)
Natural dentition	25 (78.13%)
Fixed prosthesis	4 (12.50%)
Implants	2 (6.25%)
Fixed prosthesis and implants	1 (3.13%)

*⁣* ^*∗*^Mean ± SD.

**Table 2:** Occlusal thickness and wearing time distribution by gender.

	Mean ± SD	Minimum	Maximum
Occlusal thickness (male *N* = 14)	2.89 ± 0.24 mm	1.5 mm	3.5 mm
Occlusal thickness (female *N* = 18)	2.94 ± 0.28 mm	1.5 mm	3.5 mm
Wearing time per day (male *N* = 14)	6.28±1.08 h	5.17 h	8.25 h
Wearing time per day (female *N* = 18)	7.14±1.36 h	5.50 h	12.75 h

**Table 3:** Force distribution at the initial moment at the occlusal splint placement.

Tooth	Male (*N* = 14)		Female (*N* = 18)		*t*	*p*
	Mean ± SD	Std. error mean	Mean ± SD	Std. error mean		
Right second molar (2)	11.05 ± 1.76	0.61	10.98 ± 2.12	0.68	0.32	*p* > 0.01
Right first molar (3)	12.56 ± 2.02	0.59	11.68 ± 1.81	0.54	2.04	*р*<0.05
Right second premolar (4)	8.57 ± 2.01	0.62	10.02 ± 1.21	0.48	1.89	*p* > 0.01
Right first premolar (5)	5.38 ± 2.56	0.85	5.52 ± 2.24	0.79	0.11	*p* > 0.01
Right canine (6)	4.59 ± 1.95	0.98	5.01 ± 2.02	0.84	0.33	*p* > 0.01
Right lateral incisor (7)	3.02 ± 0.96	0.45	2.76 ± 1.23	0.51	0.17	*p* > 0.01
Right central incisor (8)	1.98 ± 1.04	0.57	2.13 ± 1.58	0.65	0.17	*p* > 0.01
Left central incisor (9)	2.56 ± 1.25	0.61	3.15 ± 1.98	0.58	0.70	*p* > 0.01
Left lateral incisor (10)	3.56 ± 2.02	0.63	3.98 ± 1.76	0.62	0.16	*p* > 0.01
Left canine (11)	5.24 ± 1.53	0.45	6.01 ± 1.45	0.49	0.50	*p* > 0.01
Left first premolar (12)	6.04 ± 1.96	0.60	6.24 ± 2.01	0.73	0.22	*p* > 0.01
Left second premolar (13)	11.39 ± 1.85	0.71	10.56 ± 1.23	0.59	0.91	*p* > 0.01
Left first molar (14)	13.12 ± 1.21	0.53	12.69 ± 1.53	0.54	0.57	*p* > 0.01
Left second molar (15)	10.94 ± 0.99	0.59	9.27 ± 1.10	0.62	1.95	*p* < 0.05

**Table 4:** Force distribution 3 months after the occlusal splint placement.

Tooth	Male (*N* = 14)		Female (*N* = 18)		*t*	*p*
	Mean ± SD	Std. error mean	Mean ± SD	Std. error mean		
Right second molar (2)	10.85 ± 1.45	0.63	10.32 ± 1.84	0.62	0.33	*p* > 0.01
Right first molar (3)	11.46 ± 1.85	0.61	10.74 ± 1.78	0.59	0.86	*p* > 0.01
Right second premolar (4)	9.89 ± 1.96	0.58	9.92 ± 1.51	0.51	0.03	*p* > 0.01
Right first premolar (5)	6.12 ± 2.21	0.78	5.97 ± 2.31	0.82	0.13	*p* > 0.01
Right canine (6)	5.99 ± 1.59	0.87	5.45 ± 1.67	0.86	0.44	*p* > 0.01
Right lateral incisor (7)	3.91 ± 0.93	0.51	3.55 ± 1.43	0.42	0.54	*p* > 0.01
Right central incisor (8)	2.59 ± 1.02	0.61	2.91 ± 1.31	0.69	0.35	*p* > 0.01
Left central incisor (9)	1.92 ± 1.22	0.64	2.33 ± 1.65	0.53	0.45	*p* > 0.01
Left lateral incisor (10)	3.76 ± 1.99	0.58	3.25 ± 1.91	0.60	0.61	*p* > 0.01
Left canine (11)	6.01 ± 1.49	0.49	6.84 ± 1.28	0.38	1.35	*p* > 0.01
Left first premolar (12)	6.39 ± 1.89	0.58	7.08 ± 2.33	0.64	0.88	*p* > 0.01
Left second premolar (13)	10.44 ± 1.81	0.75	10.87 ± 1.56	0.62	0.12	*p* > 0.01
Left first molar (14)	10.65 ± 1.18	0.56	10.96 ± 1.45	0.51	0.12	*p* > 0.01
Left second molar (15)	10.02 ± 0.97	0.56	9.81 ± 1.22	0.68	0.61	*p* > 0.01

**Table 5:** Force distribution at the initial moment and 3 months after the occlusal splint placement.

Zone	Male (*N* = 14)		Female (*N* = 18)		*p* (t)
	Mean ± SD	Std. error mean	Mean ± SD	Std. error mean	
Right zone (initial moment)	47.15 ± 1,76	0.67	48.10 ± 1.74	0.64	*p* > 0.01 (1.03)
Right zone (after 3 months)	50.81 ± 1.57	0.66	48.86 ± 1.69	0.64	*p* < 0.05 (2.11)
Left zone (initial moment)	52.85 ± 1.54	0.50	51.90 ± 1.58	0.60	*p* > 0.01 (1.22)
Left zone (after 3 months)	49.19 ± 1.51	0.59	51.14 ± 1.63	0.57	*p* < 0.05 (2.38)

**Table 6:** Distribution of occlusal force in men at initial moment and 3 months after occlusal splint placement.

Zone	Male (*N* = 14)		*p* (t)
	Mean ± SD	Std. error mean	
Right zone (initial moment)	47.15 ± 1,76	0.67	*p* < 0.01 (3.89)
Right zone (after 3 months)	50.81 ± 1.57	0.66	

Left zone (initial moment)	52.85 ± 1.54	0.50	*p* < 0.01 (4.73)
Left zone (after 3 months)	49.19 ± 1.51	0.59	

**Table 7:** Distribution of occlusal force in women at initial moment and 3 months after occlusal splint placement.

Zone	Female (*N* = 18)		*p* (t)
	Mean ± SD	Std. error mean	
Right zone (initial moment)	48.10 ± 1.74	0.64	*p* > 0.01 (0.9)
Right zone (after 3 months)	48.86 ± 1.69	0.64	

Left zone (initial moment)	51.90 ± 1.58	0.60	*p* > 0.01 (0.92)
Left zone (after 3 months)	51.14 ± 1.63	0.57	

## Data Availability

The data for this research article can be obtained by contacting the corresponding author upon request.
